# Etiology and Clinical Features of Full-Term Neonatal Bacterial Meningitis: A Multicenter Retrospective Cohort Study

**DOI:** 10.3389/fped.2019.00031

**Published:** 2019-02-13

**Authors:** Min Xu, Lan Hu, Heyu Huang, Liping Wang, Jintong Tan, Yongjun Zhang, Chao Chen, Xi Zhang, Lisu Huang

**Affiliations:** ^1^Department of Neonatology, Xinhua Hospital, Shanghai Jiaotong University School of Medicine, Shanghai, China; ^2^Department of Neonatology, Children Hospital of Fudan University, Shanghai, China; ^3^Department of Pediatric Infectious Diseases, Xinhua Hospital, Shanghai Jiaotong University School of Medicine, Shanghai, China; ^4^Clinical Research Unit, Xinhua Hospital, Shanghai Jiaotong University School of Medicine, Shanghai, China

**Keywords:** pathogens, clinical features, neonatal bacterial meningitis, *Group B Streptococcus*, *Escherichia coli*, neurological complications

## Abstract

**Objective:** Neonatal bacterial meningitis is a severe infectious disease with a high risk of neurodevelopmental sequelae. The causative pathogens may be related to specific clinical features of the disease. Therefore, this study aimed at determining the pathogen-specific and clinical features of bacterial meningitis in full-term neonates.

**Methods:** We enrolled neonates from the Shanghai Neonate Meningitis Cohort (2005–2017), which is a multicenter retrospective cohort that recruits almost all full-term neonates in Shanghai who underwent lumbar puncture. Patient history and clinical examination results were extracted from the computer-documented information systems of four hospitals. The trends of pathogen distribution were analyzed and differences in the clinical manifestations, treatment, and clinical outcomes at discharge were compared according to the causative pathogen. Logistic regression was used to evaluate the pathogen-specific risk of neurological complications.

**Results:** In total, 518 cases of neonatal meningitis, including 189 proven cases, were included. *Group B Streptococcus* (GBS) and *Escherichia coli* (*E. coli*) were the leading pathogens in proven cases of early-onset and late-onset neonatal meningitis, respectively. The proportion of early-onset and late-onset GBS and late-onset *E. coli* meningitis cases increased gradually. GBS meningitis had the highest risk of neurological complications, whereas the overall incidence of hydrocephalus and brain abscess in *E. coli* was higher than that in GBS.

**Conclusions:** Rates of neonatal GBS and *E. coli* meningitis were high in 2005–2017 in Shanghai, and the risk of neurological complications was also high. Therefore, active prevention, rational use of antibiotics, and continuous monitoring of GBS and *E. coli* in neonates should be initiated in Shanghai.

## Introduction

Despite advances in infant intensive care, neonatal meningitis remains a devastating disease. In some developed countries, *Group B Streptococcus* (GBS) and *Escherichia coli* (*E. coli*) were reported as the main pathogens of bacterial meningitis in young infants (1–4). However, in the developing world, the microbiology of bacterial meningitis in neonates varies geographically. A World Health Organization-supported study in four African countries showed that *Klebsiella pneumoniae* and *E. coli* were the most-prevalent causative pathogens in cases of neonatal meningitis (5). Reports from the central and western provinces of China showed that *E. coli* was a commonly isolated bacterium in cases of neonatal meningitis; other pathogens included *Staphylococcus epidermidis, K. pneumonia*, and GBS (6, 7). However, no large-scale study has examined the etiology of neonatal bacterial meningitis in the eastern regions of China.

The morbidity rate of neonatal meningitis, especially the incidence of neurological sequelae, is currently high worldwide (8, 9), and the clinical severity and prognosis of neonatal bacterial meningitis may be associated with the type of invading pathogen. Most studies have focused on the clinical features of a single pathogen. For instance, both hyper-, and hypothermia were reported as being the most frequent clinical signs of *E. coli* meningitis at admission (10). Further, GBS meningitis, an isolated illness caused by a gram-positive pathogen, was found to be characterized by the presence of specific signs of meningitis, such as lethargy and seizure (2, 11). Symptomatically, gram-negative pathogens are more likely to cause seizures as the initial manifestation of meningitis and are associated with higher cerebrospinal fluid (CSF) white blood cell counts than gram-positive pathogens (12). However, studies on the systematic identification of the clinical features of different pathogens are lacking. Selection bias in such comparative studies can only be reduced by simultaneously comparing the clinical differences of different pathogens in the same study.

Understanding the differences in the clinical manifestations of these pathogens is important for early identification of pathogens and the rational use of antibiotics. In addition, the lack of studies examining the etiology of neonatal bacterial meningitis in the eastern regions of China impedes the development of strategies for the prevention of neonatal bacterial meningitis in these regions. Therefore, this large-scale study aimed to identify the trends in bacterial infection among cases of meningitis in full-term neonates in Shanghai and explore the pathogen-specific and clinical features of bacterial meningitis among full-term neonates.

## Materials and Methods

The Shanghai Neonate Meningitis Cohort is a multicenter retrospective cohort, including almost all full-term neonates who underwent lumbar puncture in Shanghai (2005–2017). It was conducted at neonatal wards of four tertiary class A pediatric hospitals in Shanghai that examined approximately 95% of the neonates with bacterial meningitis in Shanghai (Xinhua Hospital, affiliated to Shanghai Jiaotong University School of Medicine; Shanghai Children's Medical Center, affiliated to Shanghai Jiaotong University School of Medicine; Children's Hospital of Shanghai Jiaotong University, and Children Hospital of Fudan University).

The history and clinical examination results for all neonates were abstracted from the computer-documented hospital information systems. Approval for the study and sharing data with the coordinating institution was granted by Xinhua Hospital affiliated to Shanghai Jiaotong University School of Medicine and was consented by the other hospitals (No. XHEC-C-2017-084).

In this study, the inclusion criteria for neonatal bacterial meningitis included one or more of the following: (a) isolation of a bacterial pathogen from CSF culture; (b) isolation of the same bacterial pathogen from blood drawn simultaneously at two different sites, with CSF pleocytosis (≥20 cells/mm^3^) (13–15); and (c) no pathogen isolated from either CSF or blood, with clinical symptoms and CSF pleocytosis (≥20 cells/mm^3^). Proven meningitis was defined in patients with either inclusion criterion (a) or (b). Clinically diagnosed meningitis was defined in patients with inclusion criterion (c). Exclusion criteria were as follows: (a) gestational age < 37 weeks, (b) onset of bacterial meningitis after >28 days of life, (c) history of severe neurological disease or ventricular drain, or (d) receipt of traumatic lumbar puncture (≥10,000 × 10^6^/L red blood cells in CSF) (16) with negative CSF culture result.

Coagulase-negative staphylococci (CoNS), considered as being potential contaminants were further evaluated as follows: Three neonatologists gathered clinical data from the hospital's computerized information system, and pathogens were assessed to determine their clinical significance.

The following data were retrieved from the computer-documented hospital information systems: bacterial species, antimicrobial susceptibility testing, demographic information, clinical signs and symptoms, first laboratory examination of CSF, cranial magnetic resonance imaging (MRI) results during treatment, and clinical outcomes at discharge. Three independent neonatologists performed the data input and checked to avoid data mis-recording as much as possible.

The threshold for early-onset and late-onset diseases was seven days after birth (17, 18). Neonates presenting with symptoms of fever, lethargy, poor feeding, vomiting, cyanosis, and apnea were considered to have nonspecific symptoms. Neonates presenting with symptoms of seizures, dystonia, irritability, abnormal primitive reflexes, bulging fontanelle, and screaming were considered to have neurological symptoms. Neonates presenting with complications including omphalitis, pneumonia, skin infection, diarrhea, and urinary infection were considered to have non-neurological complications. Neurological complications were limited to abnormal cranial MRI results including ventriculitis, intracranial hemorrhage, subdural effusion, hydrocephalus, and brain abscess, which were confirmed by MRI experts. The clinical prognosis at discharge was evaluated using the Glasgow Outcome Scale (GOS) (1, death; 2, persistent vegetative state; 3, severe disability; 4, moderate disability; 5, good recovery). Moderate or severe disability was defined as any of the following conditions: spasticity, muscle weakness, and immobility in one or more limbs; microcephaly; hydrocephalus; seizure disorder; hearing loss. A GOS score of ≤ 4 was defined as poor prognosis (19). During the antibiotic treatment, some families decided to withdraw treatment. These neonates had been using antibiotics during hospitalization and did not complete antibiotic treatment before the families withdrew treatment. Once the families withdrew treatment, these neonates were discharged immediately. There were two reasons why the families withdrew treatment: (a) they can no longer afford hospitalization expenses; (b) the neonates had complications during treatment, or parents felt that the prognosis will be poor, and they lost confidence in the recovery of the neonates.

We calculated the median and interquartile range for continuous variables (as most data were not normally distributed), and frequency for categorical variables. Continuous variables were compared using nonparametric methods (Mann-Whitney *U*-test). Categorical variables were compared using the chi-square test or Fisher's exact test, where appropriate. Considering the multiple comparisons, we used the Bonferroni method to adjust for the type I error rate. Multivariate logistic regression models were used to evaluate risk factors for poor prognosis and neurological complications. All statistical analyses were performed using SPSS 17.0 (SPSS Inc, Chicago, IL, USA). The figures were produced using GraphPad Prism 6.0 (GraphPad Software, La Jolla, CA, USA).

## Results

### Pathogen Distribution

We identified 518 cases of neonatal meningitis, including 189 proven cases and 329 clinically diagnosed cases. In the proven cases, gram-positive pathogens accounted for 116 cases (61.4%), and gram-negative pathogens accounted for the remaining 73 cases (38.6%). GBS (*n* = 55, 29.1%) and *E. coli* (*n* = 55, 29.1%) were the leading pathogens in the proven cases of neonatal meningitis. Other pathogens involved in < 5 cases included CoNS (*n* = 42, 22.2%), *Enterobacteriaceae* (except *E. coli*) (*n* = 11, 5.8%), and *Enterococcus* (*n* = 10, 5.3%) (Table 1).

**Table 1:** Pathogen distribution of 189 full-term neonatal cases with proven meningitis.

**Pathogen**	**Total**	**CSF& Blood**	**CSF only**	**Blood only**
GBS	55 (29.1)	22 (43.1)	10 (21.7)	23 (25.0)
*E. coli*	55 (29.1)	20 (39.2)	13 (28.3)	22 (23.9)
CoNS	42 (22.2)	4 (7.9)	7 (15.3)	31 (33.8)
*Staphylococcus epidermidis*	21 (11.1)	1 (2.0)	1 (2.2)	19 (20.7)
*Staphylococcus haemolyticus*	3 (1.6)	0 (0.0)	1 (2.2)	2 (2.2)
*Staphylococcus hominis*	3 (1.6)	1 (2.0)	0 (0.0)	2 (2.2)
*Staphylococcus saprophyticus*	2 (1.1)	0 (0.0)	1 (2.2)	1 (1.1)
*Staphylococcus warneri*	1 (0.5)	0 (0.0)	1 (2.2)	0 (0.0)
*Staphylococcus lentus*	1 (0.5)	0 (0.0)	0 (0.0)	1 (1.1)
*Staphylococcus cohnii*	1 (0.5)	0 (0.0)	1 (2.2)	0 (0.0)
Other types	10 (5.3)	2 (3.9)	2 (4.3)	6 (6.5)
Enterobacteriaceae (except *E. coli*)	11 (5.8)	1 (2.0)	5 (10.9)	5 (5.4)
*Klebsiella pneumoniae*	7 (3.7)	1 (2.0)	2 (4.3)	4 (4.3)
*Enterobacter cloacae*	1 (0.5)	0 (0.0)	1 (2.2)	0 (0.0)
*Proteus species*	1 (0.5)	0 (0.0)	1 (2.2)	0 (0.0)
*Klebsiella oxytoca*	1 (0.5)	0 (0.0)	1 (2.2)	0 (0.0)
*Plesiomonas shigelloides*	1 (0.5)	0 (0.0)	0 (0.0)	1 (1.1)
Enterococcus	10 (5.3)	1 (2.0)	4 (8.7)	5 (5.4)
*Enterococcus faecium*	8 (4.2)	1 (2.0)	3 (6.5)	4 (4.3)
*Enterococcus faecalis*	1 (0.5)	0 (0.0)	1 (2.2)	0 (0.0)
*Enterococcus gallinarum*	1 (0.5)	0 (0.0)	0 (0.0)	1 (1.1)
*Staphylococcus aureus*	3 (1.6)	1 (2.0)	0 (0.0)	2 (2.2)
*Chryseobacterium meningosepticum*	3 (1.6)	1 (2.0)	2 (4.3)	0 (0.0)
*Listeria monocytogenes*	3 (1.6)	0 (0.0)	3 (6.5)	0 (0.0)
*Stenotrophomonas maltophilia*	2 (1.1)	0 (0.0)	1 (2.2)	1 (1.1)
*Acinetobacter bauman*	1 (0.5)	0 (0.0)	1 (2.2)	0 (0.0)
*Haemophilus influenzae*	1 (0.5)	0 (0.0)	0 (0.0)	1 (1.1)
*Bacillus subtilis*	1 (0.5)	1 (2.0)	0 (0.0)	0 (0.0)
*Streptococcus gallolyticus*	1 (0.5)	0 (0.0)	0 (0.0)	1 (1.1)
*Micrococcus luteus*	1 (0.5)	0 (0.0)	0 (0.0)	1 (1.1)
Total	189 (100.0)	51 (100.0)	46 (100.0)	92 (100.0)

*Numbers are presented as n (%). “CSF & Blood,” “CSF only,” and “Blood only” represent pathogens detected in both the CSF and blood cultures; CSF culture only; and blood culture only, respectively. E. coli, Escherichia coli; GBS, Group B Streptococcus; CSF, cerebrospinal fluid*.

### Trends in Pathogen Distribution

**Figure 1:**
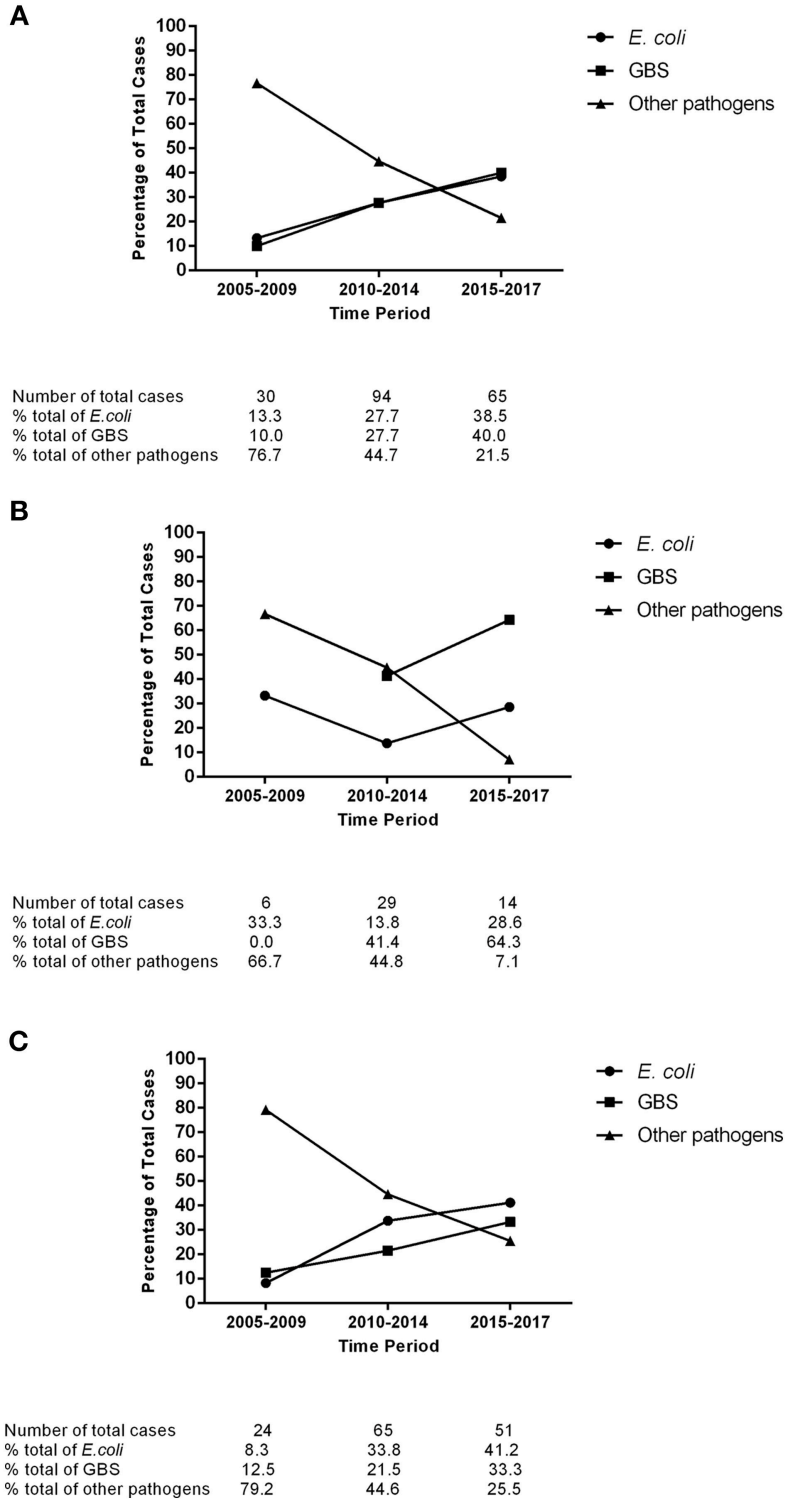
Trends in the pathogen distribution of full-term neonatal meningitis in Shanghai, 2005–2017. **(A)** All meningitis cases; **(B)** Early-onset meningitis cases; **(C)** Late-onset meningitis cases. *E. coli, Escherichia coli*; *GBS, Group B Streptococcus*.

Early-onset bacterial meningitis accounted for 31.2% of the proven cases among full-term neonates in Shanghai during 2005–2017. There was an upward trend in the proportions of both early-onset and late-onset GBS meningitis (Figure 1). Over the study duration, GBS was the leading pathogen in early-onset meningitis (21/49, 42.9%). The proportion of late-onset *E. coli* meningitis cases increased gradually, and *E. coli* was the most-common pathogen in cases of late-onset meningitis (45/140, 32.1%). The proportion of all other pathogens decreased gradually in cases of both early-onset and late-onset meningitis.

### Antibiotic Susceptibility

All GBS strains were sensitive to gentamicin, ampicillin, vancomycin, and linezolid while only 16.2% of GBS cases were sensitive to clindamycin (Table 2). Only one strain was resistant to penicillin. *E. coli* showed a high sensitivity to cefepime (88.6%) and meropenem (100.0%), a varying resistance to ampicillin, gentamicin, cefotaxime, and ceftriaxone (71.4, 36.8, 50.0, and 43.3%, respectively), and a high proportion of extended spectrum beta-lactamase (ESBL)-producing isolates (41.2%).

**Table 2:** *In vitro* antimicrobial susceptibility testing of common pathogens from full-term neonatal cases with proven meningitis.

**Organism**	**PEN**	**AMP**	**GEN**	**CLI**	**VAN**	**LNZ**	**CTX**	**CRO**	**FEP**	**MEM**	**ESBL (+)**
GBS	42/43 (97.7)	25/25 (100.0)	–	6/37 (16.2)	43/43 (100.0)	40/40 (100.0)	11/11 (100.0)	41/43 (95.3)	–	10/10 (100.0)	–
*E. coli*	–	10/35 (28.6)	24/38 (63.2)	–	–	–	19/38 (50.0)	17/30 (56.7)	31/35 (88.6)	25/25 (100.0)	7/17 (41.2)
CoNS	3/32 (9.4)	–	19/30 (63.3)	18/32 (56.3)	32/32 (100.0)	31/31 (100.0)	–	–	–	–	–
Enterobacteriaceae (except *E. coli*)	–	0/8 (0.0)	6/8 (75.0)	–	–	–	3/8 (37.5)	2/2 (100.0)	4/7 (57.1)	6/6 (100.0)	–
Enterococcus	2/4 (50.0)	4/6 (66.7)	5/6 (83.3)	–	7/7 (100.0)	6/6 (100.0)	–	–	–	–	–

*Numbers represent susceptible pathogens/pathogens tested (% susceptible). PEN, penicillin; AMP, ampicillin; GEN, gentamicin; CLI, clindamycin; VAN, vancomycin; LNZ, linezolid; CTX: cefotaxime; CRO, ceftriaxone; FEP, cefepime; MEM, meropenem; ESBL (+), extended spectrum beta-lactamase (+)*.

### Demographic Information and Clinical Symptoms

Of the 518 neonates, 307 (59.3%) were boys, 15 (2.9%) had low birth weights (< 2,500 g), and 344 (66.4%) were born by cesarean section. Fever was the predominant symptom in all cases, regardless of the etiology, and 197 cases (38.0%) showed neurological symptoms. Seizures (*n* = 83, 16.0%) were the leading presentation of neurological symptoms. Poor feeding, seizures, and irritability occurred more frequently in GBS cases than in the clinically diagnosed cases (*P* < 0.05). Incidence rates of the other symptoms did not differ among the clinically diagnosed, GBS and *E. coli* cases (Table 3).

**Table 3:** Characteristics of full-term neonatal meningitis cases caused by the three most commonly cultured pathogens.

**Characteristics**	**Clinically diagnosed cases (*n* = 329)**	**GBS cases (*n* = 55)**	***E. coli* cases (*n* = 55)**
Men, *n* (%)	203 (61.7)	28 (50.9)	27 (49.1)
Gestational age (weeks), median (IQR)	39.1 (38.2–40.0)	39.0 (38.0–40.0)	39.0 (38.0–40.1)
Birth weight (kg), median (IQR)	3.4 (3.1–3.6)	3.3 (3.1–3.5)	3.2 (3.0–3.6)
Early-onset, *n* (%)	108 (32.8)	21 (38.2)	10 (18.2)
Cesarean delivery, *n* (%)	213 (64.7)	42 (76.4)	35 (63.6)
Course of treatment (days), median (IQR)	25 (19–33)	36 (29–51)^†^^‡^	28 (22–40)
Nonspecific symptoms, *n* (%)^a^	294 (89.4)	54 (98.2)	52 (94.5)
Fever	253 (76.9)	50 (90.9)	49 (89.1)
Lethargy	95 (28.9)	24 (43.6)	22 (40.0)
Poor feeding	82 (24.9)	24 (43.6)^†^	20 (36.4)
Vomit	23 (7.0)	4 (7.3)	2 (3.6)
Cyanosis	19 (5.8)	2 (3.6)	2 (3.6)
Apnea	7 (2.1)	2 (3.6)	1 (1.8)
Neurological symptoms, *n* (%)^b^	122 (37.1)	27 (49.1)	23 (41.8)
Seizures	47 (14.3)	15 (27.3)^†^	9 (16.4)
Dystonia	36 (10.9)	12 (21.8)	9 (16.4)
Irritability	18 (5.5)	10 (18.2)^†^	6 (10.9)
Abnormal primitive reflexes	56 (17.0)	4 (7.3)	9 (16.4)
Bulging fontanelle	14 (4.3)	3 (5.5)	4 (7.3)
Screaming	1 (0.3)	1 (1.8)	2 (3.6)
**CSF findings, median (IQR)**			
WBC count (× 10^6^/L)	102 (37–420)	1111 (114–3,920)^†^	491 (79–3,248)^*^
Protein, g/L	1.3 (0.9–2.0)	2.9 (1.6–4.7)^†^	2.0 (1.5–3.3)^*^
Glucose, mmol/L	2.0 (1.1–2.4)	1.0 (0.8–2.0)^†^	1.6 (0.1–2.0)^*^
Non-neurological complications, *n* (%)^c^	176 (53.5)	27 (49.1)	21 (38.2)
Omphalitis	20 (6.1)	2 (3.6)	5 (9.1)
Pneumonia	131 (39.8)	21 (38.2)	14 (25.5)
Skin infection	5 (1.5)	0 (0.0)	2 (3.6)
Diarrhea	49 (14.9)	7 (12.7)	3 (5.5)
Urinary infection	6 (1.8)	0 (0.0)	0 (0.0)
Neurological complications, *n* (%)^d^	82 (25.9)	24 (44.4)^†^	22 (40.7)^*^
Ventriculitis	13 (4.1)	2 (3.7)	3 (5.6)
Intracranial hemorrhage	59 (18.7)	12 (22.2)	7 (13.0)
Subdural effusion	17 (5.4)	12 (22.2)^†^	9 (16.7)^*^
Hydrocephalus	17 (5.4)	2 (3.7)^‡^	10 (18.5)^*^
Brain abscess	5 (1.6)	5 (9.3)^†^	5 (9.3)^*^
Poor prognosis, *n* (%)^e^	88 (26.7)	10 (18.2)	21 (38.2)
Death, *n* (%)	2 (0.6)	0 (0.0)	0 (0.0)
Withdrew treatment, *n* (%)^f^	47 (14.3)	5 (9.1)	12 (21.8)

a *Any one or more of the symptoms including fever, lethargy, poor feeding, vomit, cyanosis, and apnea were defined as the presence of nonspecific symptoms*.

b *Any one or more of the symptoms including seizure, dystonia, irritability, abnormal primitive reflexes, bulging fontanelle, and screaming were defined as the presence of neurological symptoms*.

c *Any one or more of the complications including omphalitis, pneumonia, skin infection, diarrhea, and urinary infection were defined as the presence of non-neurological complications*.

d *Any one or more of the neurological complications including ventriculitis, intracranial hemorrhage, subdural effusion, hydrocephalus and brain abscess confirmed by cranial magnetic resonance imaging were defined as the presence of neurological complications*.

e *Glasgow Outcome Scale score of ≤ 4 is defined as poor prognosis*.

f *Families withdrew treatment due to economic difficulties and/or poor prognosis*.

* *Comparison between clinically diagnosed cases and E. coli cases (adjusted P < 0.05 by the Bonferroni method)*.

† *Comparison between clinically diagnosed cases and GBS cases (adjusted P < 0.05 by the Bonferroni method)*.

‡ *Comparison between E. coli cases and GBS cases (adjusted P < 0.05 by the Bonferroni method)*.

*IQR, interquartile range; CSF, cerebrospinal fluid; WBC, white blood cell; GBS, Group B Streptococcus; E. coli, Escherichia coli*.

### CSF Findings

Lumbar puncture was performed in all 518 cases. The GBS and *E. coli* cases showed higher CSF white blood cell counts, protein levels, and lower glucose levels than the clinically diagnosed cases (*P* < 0.05). There were no significant differences in CFS findings between the GBS and *E. coli* cases.

### Clinical Outcomes at Discharge

A total of 3 neonates died in hospital, and 140 neonates (27.0%) had poor prognosis (GOS score ≤ 4) at discharge. Seventy-six neonates (14.7%) were discharged after families chose to withdraw treatment, in which 25 neonates received >2 weeks of treatment. The rates of in-hospital deaths and treatment withdrawal did not differ significantly between the clinically diagnosed, GBS, and *E. coli* meningitis cases. There was also no difference between the clinically diagnosed group and each proven group in terms of the incidence of poor prognosis. However, the rate of poor prognosis was significantly higher in *E. coli* cases than in GBS cases (*P* < 0.05), and this difference was consistent when we further adjusted for gender, birth weight, and early or late-onset pattern in the multivariate analyses (Supplementary Table 1).

Most neonates (501/518, 96.7%) underwent cranial MRI during treatment, and the results of 146 neonates (29.1%) were abnormal, including ventriculitis (21/501, 4.2%), intracranial hemorrhage (88/501, 17.6%), subdural effusion (41/501, 8.2%), hydrocephalus (33/501, 6.6%), and brain abscess (17/501, 3.4%). Neonates with GBS meningitis had a much higher risk of total neurological complications, especially subdural effusion and brain abscess, than clinically diagnosed cases (total neurological complications: odds ratio [OR], 2.3; 95% confidence interval [CI], 1.3–4.1; subdural effusion: OR, 5.0; 95% CI, 2.2–11.3; and brain abscess: OR, 6.3; 95% CI, 1.8–22.8). Neonates with *E. coli* meningitis had a relatively higher risk of total neurological complications, subdural effusion, hydrocephalus, and brain abscess than the clinically diagnosed cases (total neurological complications: OR, 2.0; 95% CI, 1.1–3.6; subdural effusion: OR, 3.5; 95% CI, 1.5–8.4; hydrocephalus: OR, 4.0; 95% CI, 1.7–9.3; and brain abscess: OR, 6.3; 95% CI, 1.8–22.8). Compared to neonates with GBS meningitis, those with *E. coli* meningitis showed a higher overall incidence of hydrocephalus and brain abscess.

Multivariate analysis of the neurological complications showed that neonates with GBS and *E. coli* meningitis had a significantly higher risk of neurological complications than those with clinically diagnosed meningitis (GBS: OR, 2.3; 95% CI, 1.3–4.3; *E. coli*: OR, 2.0; 95% CI, 1.1–3.6). There was no significant difference in the risk of neurological complications between the clinically diagnosed cases and the cases of the other pathogens (Table 4).

**Table 4:** Univariate and multivariate analyses of risk factors for neurological complications in full-term neonatal meningitis cases.

**Variables**	**Univariate analysis**		**Multivariate analysis**	
	**OR (95% CI)**	***P***	**OR (95% CI)**	***P***
Men vs. women	1.1 (0.8–1.7)	0.55	1.2 (0.8–1.8)	0.43
Birth weight < 2,500 g vs. ≥2,500 g	0.7 (0.2–2.6)	0.60	0.7 (0.2–2.7)	0.61
Early-onset vs. Late-onset	1.1 (0.7–1.7)	0.62	1.1 (0.7–1.7)	0.75
**PATHOGEN**				
Clinically diagnosed cases	Reference		Reference	
*E. coli* cases	2.0 (1.1–3.6)	0.03	2.0 (1.1–3.6)	0.03
GBS cases	2.3 (1.3–4.1)	0.006	2.3 (1.3–4.3)	0.005
Cases of other pathogens	0.9 (0.5–1.6)	0.64	0.9 (0.5–1.6)	0.74

*GBS, Group B Streptococcus; E. coli, Escherichia coli; OR, odds ratio; CI, confidence interval*.

## Discussion

To the best of our knowledge, this is the first long-term study of full-term neonatal meningitis with a large sample size in Shanghai, China. We found that GBS and *E. coli* were the leading pathogens in proven cases of neonatal meningitis, which was similar with that of the developed countries. GBS and *E. coli* were the most-common pathogens in early-onset and late-onset neonatal meningitis, respectively, both of which showed an increasing trend and had a high risk of neurological complications.

In some developed countries and regions, GBS is the most-prevalent pathogen causing meningitis in young infants, and is confined to this population (1, 2, 4). In the USA, approximately 16.7–22.7% of pregnant women showed GBS colonization (20). GBS prevention activities increased since the 1990s, and the first recommendation for universal screening was issued in 2002. This resulted in an estimated 80% decrease in early-onset GBS infection in the USA (21). In China, GBS infection occurs in 7.1–10.4% of pregnant women, a lower prevalence than that of the USA (22, 23). However, in our study, GBS was the leading pathogen in early-onset meningitis, and the prevalence of both early-onset and late-onset GBS meningitis has shown an increasing trend in recent years. Regarding antimicrobial susceptibility, neonates with GBS meningitis showed high sensitivity to penicillin and ampicillin, which remain the agents of choice for intrapartum antibiotic prophylaxis (IAP) (24, 25). These results may be partially related to the non-wide implementation of IAP in China.

In this study, neonates with GBS meningitis had a significantly higher incidence of poor feeding, seizures, and irritability than neonates with clinically diagnosed meningitis. In addition, the incidence of nonspecific and neurological symptoms of GBS and *E. coli* meningitis was both slightly higher than those of clinically diagnosed meningitis (although not significantly). As for the CSF findings, GBS and *E. coli* meningitis were significantly more serious than clinically diagnosed meningitis. Based on these, if a newborn presents with several clinical symptoms combined with severe CSF findings, we should be wary of GBS or *E. coli* meningitis.

The fact that neonatal GBS meningitis had the highest risk of neurological complications may be related to the virulence of GBS and the severe damage to the brain caused by its infection. *In vitro* and clinical studies have found that GBS can promote blood-brain barrier penetration (26–28) and may cause severe cerebrovascular diseases (29). Abnormal findings on full-term neonatal MRI were associated with a poor neurodevelopmental outcome (30). A meta-analysis on neurodevelopmental impairment after GBS disease in infants aged < 90 days showed that GBS meningitis was an important risk factor for moderate-to-severe neurodevelopmental impairment, affecting approximately 20% of survivors (31). Therefore, it is important to conduct brain imaging on neonates with GBS meningitis and to follow-up for long-term neurological sequelae. In addition, the implementation of IAP may be associated with a low mortality in cases of early-onset GBS disease, and mild late-onset GBS disease (32, 33). Currently, in China, only a few hospitals perform IAP by strictly following a risk-based strategy. Hence, IAP should be promoted and continuous monitoring of GBS should be initiated in China.

Similar to GBS, we also found that *E. coli* meningitis had a significantly higher risk of neurological complications than those of clinically diagnosed meningitis, especially subdural effusion, hydrocephalus, and brain abscess. As reported in other studies, neonatal *E. coli* meningitis may cause learning and memory impairments in adulthood (34), which may be related to the high incidence of *E. coli* neurological complications. Although *E. coli* meningitis had a similar incidence of neurological complications to GBS, the overall incidence of hydrocephalus and brain abscess was higher than the latter. As serious neurological complications, hydrocephalus and brain abscess were associated with significant long-term morbidity and mortality (35, 36). We further speculate that the high proportion of ESBL-producing isolates in *E. coli* cases increases the risk of serious neurological complications. The high proportion of ESBL-producing isolates in *E. coli* cases is similar to that reported in a large multicenter study in China, which may be related to the universal antibiotic overuse or misuse in China (37). At present, there is no national guideline for the treatment of neonatal meningitis in China, so the choice and course of antimicrobial treatment may vary in different hospitals. Therefore, a consensus guideline for treatment of neonatal meningitis should be established and better monitoring of drug-resistant bacteria should be encouraged.

To our knowledge, this is the first large-scale study of neonatal meningitis in Shanghai investigating pathogen trends in the past 13 years, and its results have furthered the understanding of the pathogen-specific and clinical features of bacterial meningitis in full-term neonates. However, there are some limitations. First, the meningitis cases in our study were diagnosed based on CSF pleocytosis, but the interpretation of CSF pleocytosis parameters are not yet standardized (38). This, together with the exclusion of infants who received traumatic lumbar punctures, may have led to underdiagnoses of meningitis in some cases and overdiagnoses in others, limiting this study's ability to estimate of overall burden of neonatal meningitis in Shanghai. Second, viral studies were not routinely considered without significant clinical signs and symptoms in neonates. However, CSF leukocytosis may also occur in neonatal viral meningitis. For example, a proportion of neonatal enteroviral meningitis can cause CSF leukocytosis (39, 40), thus the incidence of neonatal viral meningitis has likely been underestimated. Further, intracranial hemorrhage might also lead to CSF leukocytosis and be confused with clinically diagnosed cases. Third, although CoNS is sometimes a contaminant, it has increasingly been recognized as a cause of clinically significant nosocomial bloodstream infections, particularly in neonates (41). As a result, we did not rule out CoNS cases, which might introduce misdiagnosis; we only included cases with typical meningitis symptoms and signs, in addition to those with positive CSF culture or positive blood culture from blood drawn simultaneously at two different sites. Fourth, some families withdrew treatment, which increased the lost to follow-up rate. This might have introduced bias to the results, such as the incidence of neurological complications. To follow-up on this hospital-based retrospective survey, we plan to address these limitations by conducting a more detailed prospective study on neonatal meningitis in the future.

Rates of neonatal GBS and *E. coli* meningitis were high in 2005–2017 in Shanghai, and the risk of neurological complications was also high. Therefore, active prevention, rational use of antibiotics and continuous monitoring of GBS and *E. coli* in neonates should be initiated in Shanghai.

## Data Availability Statement

The datasets for this manuscript are not publicly available because: part of the data is included in other manuscripts under preparation. Requests to access the datasets should be directed to Lisu Huang: huanglisu@xinhuamed.com.cn.

## Author Contributions

MX, LaH, YZ, CC, XZ, and LiH conceptualized and designed the study. HH, LW, and JT collected the data. MX, YZ, XZ, and LiH analyzed the data. MX drafted the initial manuscript. All authors contributed to the interpretation of the data and contributed to the final draft of the manuscript.

### Conflict of Interest Statement

The authors declare that the research was conducted in the absence of any commercial or financial relationships that could be construed as a potential conflict of interest.

## Abbreviations

CoNS: Coagulase-negative staphylococci
*E. coli*: *Escherichia coli*
ESBL: extended spectrum beta-lactamase
GBS: *Group B Streptococcus*
GOS: Glasgow Outcome Scale
IAP: intrapartum antibiotic prophylaxis
CSF: cerebrospinal fluid.
