# Supplementary material for: Etiology and Clinical Features of Full-Term Neonatal Bacterial Meningitis: A Multicenter Retrospective Cohort Study
Source: Front Pediatr. 2019 Feb 13;7:31. doi: 10.3389/fped.2019.00031 (PMC6381005; doi:10.3389/fped.2019.00031)
Supplement: Supplementary file 1 [file Table_1.docx]

Supplementary Material

**SUPPLEMENTARY TABLE 1 |** Univariate and multivariate analyses of risk factors for poor prognosis in full-term neonatal meningitis cases.

| **Variables** | **Univariate Analysis** | | **Multivariate Analysis** | |
| --- | --- | --- | --- | --- |
|  | **OR （95% CI）** | ***P*** | **OR （95% CI）** | ***P*** |
| Men vs. women | 0.8 (0.5-1.1) | 0.16 | 0.8 (0.5-1.2) | 0.22 |
| Birth weight < 2500 g vs. ≥2500 g | 1.8 (0.6-5.3) | 0.26 | 1.7 (0.6-5.0) | 0.30 |
| Early-onset vs. Late-onset | 0.6 (0.4-1.0) | 0.04 | 0.7 (0.4-1.0) | 0.07 |
| Pathogen |  |  |  |  |
| Clinically diagnosed cases | Reference |  | Reference |  |
| *E. coli* cases | 1.7 (0.9-3.1) | 0.08 | 1.6 (0.9-3.0) | 0.11 |
| GBS cases | 0.6 (0.3-1.3) | 0.18 | 0.6 (0.3-1.3) | 0.20 |
| Cases of other pathogens | 1.0 (0.6-1.7) | 0.98 | 0.9 (0.5-1.6) | 0.66 |

*GBS, Group B Streptococcus; E. coli, Escherichia coli; OR, odds ratio; CI, confidence interval*
